# Platelet Membrane-Coated Nanocarriers Targeting Plaques to Deliver Anti-CD47 Antibody for Atherosclerotic Therapy

**DOI:** 10.34133/2022/9845459

**Published:** 2022-01-17

**Authors:** Liang Chen, Zhongyi Zhou, Cheng Hu, Manfred F. Maitz, Li Yang, Rifang Luo, Yunbing Wang

**Affiliations:** ^1^National Engineering Research Center for Biomaterials, Sichuan University, Chengdu 610065, China; ^2^Max Bergmann Center of Biomaterials, Leibniz Institute of Polymer Research Dresden, Dresden 01069, Germany; ^3^Key Lab. for Advanced Technologies of Materials, Ministry of Education, School of Material Science and Engineering, Southwest Jiaotong University, Chengdu 610031, China

## Abstract

Atherosclerosis, the principle cause of cardiovascular disease (CVD) worldwide, is mainly characterized by the pathological accumulation of diseased vascular cells and apoptotic cellular debris. Atherogenesis is associated with the upregulation of CD47, a key antiphagocytic molecule that is known to render malignant cells resistant to programmed cell removal, or “efferocytosis.” Here, we have developed platelet membrane-coated mesoporous silicon nanoparticles (PMSN) as a drug delivery system to target atherosclerotic plaques with the delivery of an anti-CD47 antibody. Briefly, the cell membrane coat prolonged the circulation of the particles by evading the immune recognition and provided an affinity to plaques and atherosclerotic sites. The anti-CD47 antibody then normalized the clearance of diseased vascular tissue and further ameliorated atherosclerosis by blocking CD47. In an atherosclerosis model established in ApoE^−/−^ mice, PMSN encapsulating anti-CD47 antibody delivery significantly promoted the efferocytosis of necrotic cells in plaques. Clearing the necrotic cells greatly reduced the atherosclerotic plaque area and stabilized the plaques reducing the risk of plaque rupture and advanced thrombosis. Overall, this study demonstrated the therapeutic advantages of PMSN encapsulating anti-CD47 antibodies for atherosclerosis therapy, which holds considerable promise as a new targeted drug delivery platform for efficient therapy of atherosclerosis.

## 1. Introduction

Atherosclerosis, the dominant pathological basis for the occurrence and development of cardiovascular disease, is the leading cause of death worldwide [1–3]. Although the pathogenesis is still not fully clarified, Kojima et al. provide a mechanism that could explain why some plaques become clinically dangerous [4]. A key feature of the plaques is the necrotic core, which contains dead cells that have undergone a type of cell death known as necrosis. Billions of cells die every day through a process called apoptosis, which initially prevents the rupture of cell membrane and leakage of inflammatory cellular components [5–7]. Apoptotic cells are rapidly and safely removed by an evolutionarily conserved process called efferocytosis, in which the apoptotic cell is internalized and destroyed by an engulfing phagocyte before the cell membrane rupture [8].

One of the possible reasons why dying cells in plaques undergo necrosis, yet not apoptosis, might be associated with the expression of the CD47 protein, which belongs to the “do not-eat-me” molecule family that signals through the Signal-regulatory protein *α* (SIRP*α*) receptor on phagocytes to inhibit apoptotic-cell engulfment [9]. Do the dying cells in the atherosclerotic plaques undergo efferocytosis? Kojima et al. found that the “don't eat me” signal protein CD47 was expressed on the surface of remaining necrotic macrophages and vascular smooth muscle cells in the histological sections of human and murine plaques. CD47 protein can make phagocytic cells misattribute apoptotic cells as healthy cells and then escape phagocytosis. Therefore, many necrotic cells accumulate in the atherosclerotic plaques, leading to the growth of the atherosclerotic plaques. Removal of the accumulated necrotic cells in the plaque is of great significance for the treatment of atherosclerosis. Restoring the ability of phagocytic cells to recognize, phagocytize, and clear necrotic cells from the source appears as a promising and worthy direction in the treatment of atherosclerosis.

In recent years, the mechanisms of initial and advanced atherosclerosis gained increasing attention [10–12]. In a mouse model of atherosclerosis, Kojima et al. injected anti-CD47 antibodies and demonstrated an improved efferocytosis in the plaque and reduced the formation of a necrotic core. Therefore, CD47 protein was identified as a new target for the prevention and treatment of atherosclerosis. Currently, anti-CD47 antibody injection therapy also is in early clinical trials as a cancer treatment and faced many challenges [13]. The main side effect of anti-CD47 antibody therapy is anemia (a decrease in the number of red blood cells), as the high level of CD47 protein expression on the surface of red blood cells (RBCs) prevents them from preterm elimination; conversely, RBCs under CD47 therapy get consumed [14–17]. Therefore, developing a targeted drug delivery system is mandatory to promote the clinical application of the anti-CD47 antibody.

With the rapid progress in materials and chemistry, nanoparticles- (NPs-) mediated drug delivery systems have achieved considerable improvement but still are difficult to obtain clinical approval [18–23]. Conventional nanoparticle-based drug delivery systems have shortcomings that they rapidly undergo immune clearance, and they are not sufficiently specific for the targeted lesion, exhibiting poor performance in therapeutic efficacy, pharmacokinetics, and biocompatibility [24–27]. In many previous studies, synthetic hydrophilic and flexible polymers, such as polyethylene glycol (PEG), have been used as a camouflage surface modification of the nanoparticles. Due to the high flexibility and hydrophilicity of PEG, the coating forms a hydration layer, which reduces the protein adsorption on the surface, avoids immune recognition and clearance, and thus prolongs the blood circulation time [28–30]. Although some PEG-modified nanomaterials showed clinical benefit, there are growing concerns about the immune system's response to synthetic polymers, as well as the formation of antibodies against PEG, impairing their performance in long-term therapy [31, 32].

More recently, membrane-coated nanomedicines have been developed for applications like detoxification, vaccination, cardiovascular disease, and cancer treatment [33, 34]. They have benefits because they possess high biocompatibility and prolonged half-life in circulation, as well as disease-specific targeting [35–37]. Blood platelets play an important role in the initiation and development of atherosclerosis, especially in the late thrombotic complications on erosion or rupture of a vulnerable plaque [38, 39]. Local inflammation of atherosclerosis leads to endothelial activation and stimulates platelet adhesion and aggregation [40, 41]. Platelets accumulate not only in advanced plaques but also in stable plaques. In the late thrombotic complications on erosion or rupture of a vulnerable plaque, platelets play an important role. However, local inflammation of stable plaques also leads to endothelial activation and stimulates platelet attachment and aggregation. Erosion or disruption of the fibrous cap exposes thrombogenic matrix proteins that promote local platelet recruitment and aggregation. These processes involve platelet interactions with inflamed endothelial cells and the ECM components exposed to blood including fibrillary collagen, fibrin, and fibronectin, which are mediated by adhesive moieties on platelet surface. All these phenomena indicate that platelets have inherent affinity to plaques and can naturally home to atherosclerotic lesions. All these phenomena indicate that platelets have an inherent affinity to plaques and can naturally home to atherosclerotic lesions. Mimicking the inherent adhesive function of platelets can be a powerful approach for targeting plaques with high efficacy [42, 43].

**Figure 1:**
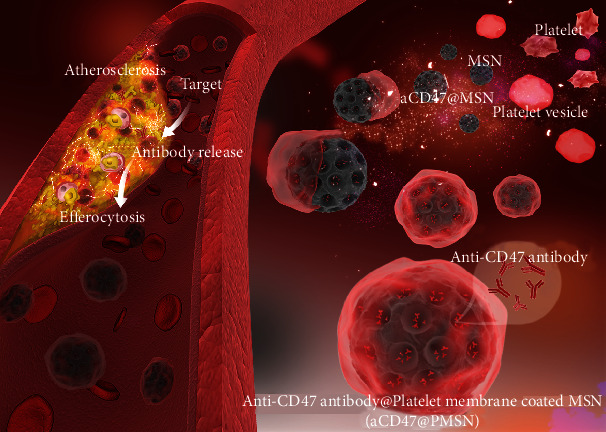
Illustration of platelet membrane-coated mesoporous silicon nanoparticles target plaques to deliver CD47 antibody for atherosclerotic treatment.

Based on these considerations, platelet membrane-coated mesoporous silicon nanoparticles (PMSN) were explored as a drug delivery system for targeting atherosclerotic plaques, making use of platelets' inherent adhesion to atherosclerosis lesions [44]. Briefly, anti-CD47 antibody was loaded in the mesoporous silicon nanoparticles, which were then wrapped in platelet membranes with the retention of almost all membrane proteins, including CD47 after PMSN preparation. Within the biomimetic modification of platelet membranes, the extended circulation time was expected by avoiding the recognition by the immune system. The atherosclerosis model of ApoE^−/−^ mice was used to confirm the targeting and accumulation specificity in atherosclerotic plaques. The anti-CD47 antibodies loaded PMSN (aCD47@PMSN) were expected to promote the efferocytosis of necrotic cells in plaques, clearing the necrotic cells in the plaques, reducing the plaque area and stabilizing the plaques, and thus reducing the risk of plaque rupture and advanced thrombosis (Figure 1).

## 2. Experimental Section

### 2.1. Materials

Cetyltrimethylammonium chloride (CTAC) solution, triethanolamine (TEA), chlorobenzene, tetraethyl orthosilicate (TEOS, >98%), 3-aminopropyltriethoxysilane (APTES), Rhodamine B isothiocyanate (RITC), and 4,6-diamidine-2-phenylindole (DAPI) were purchased from Sigma-Aldrich. InVivoMAb anti-mouse/human/rat CD47 (MIAP410, BioXcell), IgG1 (MOPC-21, BioXcell), Prostaglandin E1 (Kedechemical), Protease inhibitor tablets, EDTA, Cell Tracker Green, and Cell Tracker Deep Red were purchased from Thermo Fisher. Cell total protein extraction kits and DiO were purchased from Beyotime. Anti-CD68 antibody, anti-*α*-SMA antibody, and anti-CD31 antibody were purchased from Abcam. The mouse total ELISA kit was purchased from Multi Sciences (China).

### 2.2. Preparation of Mesoporous Silicon Nanoparticle (MSN)

We obtained MSN materials from Xi'an ruixi Biological Technology Co., Ltd. The preparation process is as follows: The water phase (0.2 g CTAB, 6 mL ethanol, 30 *μ*L TEA, and 25 mL ultrapure water) was stirred at 60°C for 0.5 h. Then, 2 mL TEOS was added to the bottom of water phase. The mixture was stirred at 500 rpm under a temperature of 60°C for 40 min. The solid samples were centrifuged at 12000 rpm for 10 min and washed with ethanol and ethanol, respectively, for three times; the final products were obtained after drying.

### 2.3. Preparation of Anti-CD47 Antibody Loaded MSN (aCD47@MSN)

The schematic illustration of the preparation process was shown in Supporting Information (Figure S1). In tube A, the final product-A aCD47@MSN was prepared by the following steps. 1 mL of 1 mg/mL of anti-CD47 antibody solution was mixed with 1 mL of 2 mg mL^−1^ of mesoporous silicon nanoparticle and then vibrated at room temperature with gentle shaking for 24 h and subjected to centrifugation. The final product-A aCD47@MSN was obtained by centrifugation at 12000 rpm for 10 min. After centrifugation, the supernatant was extracted to measure the residual antibody concentration.

### 2.4. Preparation of Platelet Membrane Vesicles

Human blood was legally obtained from Chengdu blood center and was allowed to use by The Ethics Committee of Sichuan University. The platelets were collected from platelet rich plasma (PRP) as described previously [32]. Platelet membrane vesicles were prepared by a repeated freeze-thaw process. After three repeated washes with PBS solution contained protease inhibitor, the pelleted platelet membrane vesicles were suspended in water and sonicated for 5 min using a Fisher Scientific FS30D bath sonicator (42 kHz, 100 W).

### 2.5. Preparation of Platelet Membrane Cloaking CD47@MSN (CD47@PMSN)

The schematic illustration of the preparation process was shown in Supporting Information (Figure S1). In the tube B, the final product-B was prepared by the following steps. At first, 1 mL of 1 mg/mL of anti-CD47 antibody solution was mixed with 1 mL of 2 mg mL^−1^ of mesoporous silicon nanoparticle in the tube B, then the mixed solution vibrated at room temperature with gentle shaking for 24 h. Next, platelet membrane vesicle solution was added into the above solution; platelet membrane cloaking mesoporous silicon nanoparticle aCD47@PMSN was prepared by dispersing and fusing platelet membrane vesicles with aCD47@MSN by sonication using an FS30D bath sonicator at frequency of 42 kHz and a power of 100 W for 2 min. The final product-B aCD47@PMSN was obtained by centrifugation at 12000 rpm for 10 min. After centrifugation, the supernatant was extracted to measure the residual antibody concentration.

### 2.6. Characterization of Nanoparticles

The size and zeta potential of platelet membrane vesicles, CD47@MSN and CD47@PMSN, were measured by Malvern Zetasizer ZS unit. The morphology of platelet vesicles, CD47@MSN and CD47@PMSN, was visually observed using a transmission electron microscope at 200 kV.

### 2.7. Characterization of Proteins

We used the cell total protein extraction kits (Beyotime) to extract the membrane protein of platelet vesicles and CD47@PMSN. The extracted membrane proteins were run on 12% SDS-PAGE gel in a running buffer using BIO-RAD electrophoresis system at 150 V for 1 h, and the resulting polyacrylamide gel was stained in Coomassie blue overnight for visualization. In addition, the key protein CD47 on the platelet membrane was analyzed by western blotting (WB).

### 2.8. Colocalization Study

0.2 g MSN was added into 30 mL toluene; then, 0.19 mL APTES was added to reflux at 110°C for 20 h. The product (MSN-NH_2_) was collected by centrifugation and washing with ethanol for 3 times. Then, 1 mg of RITC was added into the suspension of absolute ethanol containing 10 mg MSN-NH_2_ followed by stirring for 24 h in the dark. The product was collected by centrifugation and thoroughly washed with ethanol until the supernatant was colorless. Then, product was dried at room temperature overnight and denoted as MSN-RITC. And we labeled the platelet membrane using DiO. We then incubated RAW264.7 with these dual-fluorophore-labeled PMSN. After incubation for an additional 2 h, the cells were washed with PBS three times and fixed with paraformaldehyde (4% in PBS) for 30 min at room temperature; then, the cells were stained with DAPI. The resulting fluorescent images were detected by CLSM.

### 2.9. Antibody Loading and In Vitro Release Study

To evaluate the CD47 antibody loading capacity of MSN, the supernatant was collected, and the residual protein concentration was measured by the Mouse total ELISA kit (Multi sciences). The encapsulation efficiency (EE) and loading capacity (LC) of PMSN were calculated using the following formulae:

(1) $$\begin{array}{c} \text{Loading content }\left(\%\right)=\frac{\text{weight of loaded antibody}}{\text{weight of nanoparticles}}\times 100\%,\\ \text{Loading efficiency }\left(\%\right)=\frac{\text{weight of loaded antibody}}{\text{weight of antibody in feed}}\times 100\%. \end{array}$$

In order to study the dynamics of antibody release in vitro, the determined concentration of aCD47@MSN and aCD47@PMSN solution was added to the 20 mL PBS (pH = 7.4) solution, respectively, and continuously oscillated at 50 RPM at 37°C. In selected different time point (4, 6, 8, 12, 24, 36, 48, 60, and 72 h), 3 mL antibody release solution was removed; then, 3 mL fresh PBS solution was added. Anti-CD47 antibody concentrations of antibody release solution were measured by Mouse total ELISA kit (Multi sciences).

### 2.10. Nanoparticles Uptake by Macrophages

5 × 10^4^ RAW 264.7 cells were seeded in 24 well plates in 1 mL of DMEM medium and cultured for 24 h. The next day, 5 *μ*g of MSN-RITC, PMSN-RITC, and aCD47@PMSN-RITC was added. We fixed the cell in the well with paraformaldehyde (4% in PBS) at different times (2, 4, 6, 8, and 12 h); then, we stained the cell with DAPI. The resulting fluorescent images were observed by CLSM.

### 2.11. In Vitro PMSN Adherence to Injured Femoral Artery of the Rabbit

The injured femoral artery of the rabbit is connected to the catheter at both ends of the peristaltic pump, forming a closed loop system. Different shear stresses (4 and 8 dyne/cm^2^) are achieved by adjusting flow rates of PBS solution through peristaltic pumps. A certain volume of PMSN-RITC and MSN-RITC nanoparticles was injected into the circulatory system, and femoral artery was removed after 24 hours of circulation. Then, Ex Vivo FL Imaging and fluorescent tissue section staining (DAPI, CD31) were performed on the femoral artery.

### 2.12. In Vivo Pharmacokinetics Study

All animal experiments were carried out in accordance with The Ethics Committee of Sichuan University. Adult male BALB/c mice were selected as the in vivo model to study the half-life of aCD47@MSN-RITC and aCD47@PMSN-RITC in circulation; 200 *μ*L of aCD47@MSN-RITC and aCD47@PMSN-RITC was injected into the mice through the tail vein. The blood sample was collected at different time points after injection (0.25, 0.5, 1, 2, 4, 6, 8, 12, 24, 36, 48, 60, and 72 h) and was added into 96-well plates with 40 *μ*L PBS contained 0.2 × 10^−3^ M EDTA_2_K. The fluorescence intensity was measured by fluorescence microplate reader.

### 2.13. Accumulation of Nanoparticles in Atherosclerotic Plaques of ApoE^−/−^ Mice

After 8 weeks of western-type diets, atherosclerosis in the ApoE^−/−^ mice was detected by ultrasound test. 200 *μ*L aCD47@MSN-RITC or aCD47@PMSN-RITC was injected to the ApoE^−/−^ mice with atherosclerotic plaque through the tail vein. After 4 h, mice were sacrificed and perfused with PBS containing 4% paraformaldehyde and heparin sodium. Arterial trees and other major organs (heart, lung, spleen, liver, and kidney) were harvested and washed with PBS; the ex vivo imaging was captured using the CRI Maestro Imaging System (Cambridge Research & Instrumentation, Inc., USA). Furthermore, the frozen section of atherosclerotic plaques was stained with DAPI and Oil Red to invest the accumulation of aCD47@MSN-RITC and aCD47@PMSN-RITC.

### 2.14. Efferocytosis Assay

We labelled the SMCs with Cell Tracker Green and labelled the mouse macrophages with Cell Tracker Deep Red. Then, SMCs were treated with 50 *μ*g mL^−1^ of oxLDL for different time (0 h, 24 h, 72 h) in 96-well plates; then, we added serum free medium with different materials (IgG1, anti-CD47 antibody, aCD47@PMSN) and cocultured for 30 min at 37°C. After 30 min, macrophages were added to each well and coincubated for 2 h in serum-free medium, then analyzed using BD FACSCalibur (BD Biosciences, Mountain View, CA). Dead cells were excluded from the analysis by staining with DAPI.

### 2.15. Treatment of Atherosclerosis in ApoE^−/−^ Mice

Then, the ApoE^−/−^ mice were randomized into four groups (6 mice per group). The aCD47 group, the aCD47@MSN group, and the aCD47@PMSN group were injected with 200 *μ*L aCD47, aCD47@MSN, and aCD47@PMSN every 3 days (the concentration of aCD47 was 1 mg mL^−1^), and the control group was given 200 *μ*L of PBS every 3 days. Treatment continued for 42 days in all groups, while the mice continued on the western diet. The body weight of mice was monitored during the treatment.

### 2.16. Histology and Immunohistochemistry Analysis

At the end of the treatment, the aortas were harvested from the ApoE^−/−^ mice and fixed with paraformaldehyde (4% in PBS), then were prepared to frozen sections. For quantitative analysis of atherosclerotic plaque areas, the sections were stained with hematoxylin-eosin (H&E) and Oil Red O (ORO). For immunohistochemistry analysis, the sections stained with anti-CD68 antibody to evaluate the density of macrophages. For endothelial cells (ECs) and smooth muscle cells (SMCs), the sections were stained with anti-*α*-SMA antibody and anti-CD31 antibody. Sections of the main organs including the heart, liver, spleen, lung, and kidney were also analyzed by hematoxylin-eosin (HE) staining to evaluate the biocompatibility of PMSN.

## 3. Results and Discussion

### 3.1. Fabrication and Characterization of aCD47@PMSN

**Figure 2:**
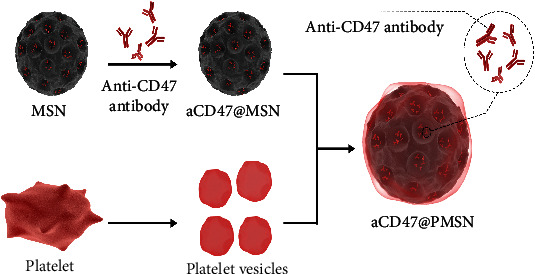
Illustration of platelet membrane-coated mesoporous silicon nanoparticle fabrication process.

To effectively deliver anti-CD47 antibody to atherosclerotic plaques, the design of the nanoparticles (NPs) in this study had to fulfill four principles: (i) NPs target atherosclerotic plaques [32, 45]; (ii) long circulating half-life to facilitate localization and plaque accumulation [45]; (iii) small size to facilitate the permeation of the endothelium, that is, NPs that are ~100 nm or smaller can penetrate the vascular wall by enhanced permeability and retention; [46, 47] (iv) controlled extracellular cargo release to enable the anti-CD47 antibody to block its target on the surface of necrotic cells at an optimal concentration and for a maximal duration. In this study, we synthesized a platelet-like delivery platform to target atherosclerotic plaques by fusing platelet membranes onto the surface of mesoporous silica nanoparticles (MSN) (Figure 2 and Figure S1).

**Figure 3:**
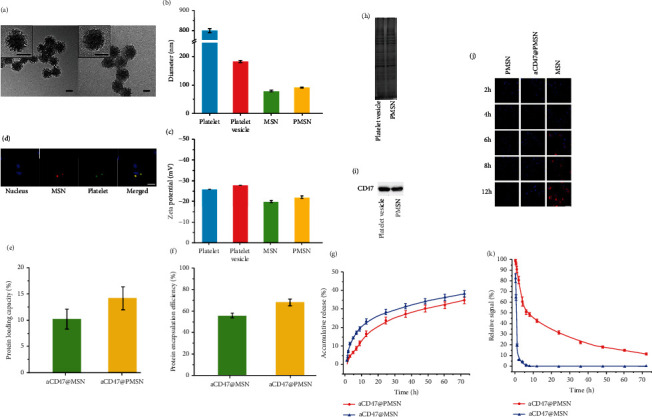
(a) Morphology of nanoparticles observed by TEM. Left: MSN; right: PMSN (scale bar = 50 nm). (b) Particle size of platelet, platelet vesicle, MSN, and PMSN measured by DLS. (c) Zeta potential of platelet, platelet vesicle, MSN, and PMSN. (d) CLSM images of PMSN internalization by cells (used for localizing platelet and MSN), the nucleus (blue), platelet shell (green), and MSN core (red) (scale bar = 25 *μ*m). (e) The anti-CD47 antibody loading capacity and (f) encapsulation efficiency in aCD47@MSN and aCD47@PMSN (*n* = 3). (g) In vitro anti-CD47 antibody release in aCD47@PMSN and aCD47@MSN. (h) Proteins in platelet vesicles and PMSN were characterized by polyacrylamide gel electrophoresis. (i) Western blot analysis of CD47 in platelet vesicles and PMSN. (j) CLSM images of MSN-RITC, PMSN-RITC, and aCD47@PMSN-RITC phagocytosed by RAW264.7 macrophages at different time points (scale bar = 50 *μ*m). (k) Pharmacokinetic studies of aCD47@MSN and aCD47@PMSN in adult male BALB/c mice.

The morphologies of MSN and PMSN were observed by TEM.  Figure 3(a) clearly shows the mesoporous structure of the MSN. The average pore diameter was 8.22 nm, which is the most suitable for protein loading, and the pore size distribution of MSN could be seen in Figure S2 [48]. After coating with the platelet membrane (Fig S3), PMSN presented a clear core-shell nanostructure; a membrane structure can be seen on the outermost layer of PMSN, indicating that MSN was successfully wrapped with the platelet membrane, with MSN as the core and the platelet membrane as the shell (Fig S4 and Fig S5). The hydrodynamic diameters (Dh) of platelet, platelet vesicles, MSN, and PMSN were determined by dynamic light scattering (DLS). As shown in Figure 3(b), coating with platelet vesicles increased the mean Dh from 80 nm (MSN, PDI: 0.087) to 93 nm (PMSN, PDI: 0.184). In addition, surface zeta potential analysis showed that PMSN had a zeta potential of −22 ± 0.7 mV, whereas platelet vesicles (−28 ± 0.9 mV) and MSN (−20.0 ± 1.1 mV) presented similar values (Figure 3(c)). To further verify the integrity of the core–shell nanoparticles, MSN were labelled with red fluorescent molecule (RITC (Rhodamine B isothiocyanate) using postgrafting method), and platelet vesicles were labeled using DiO (3,3′-dioctadecyloxacarbocyanine perchlorate). The resulting dual-fluorophore-labeled nanoparticles were incubated with endothelial cells (ECs) for 6 h and visualized using CLSM (confocal laser scanning microscopy). Resulting images showed the two fluorescent signals coincide perfectly, indicating an intact “core–shell” structure (Figure 3(d)).

The anti-CD47 antibodies are physical adsorption in the pore cavity. The driving force is hydrophobic force, hydrogen bond force, and electrostatic force. The loading capacity (LC) of aCD47@MSN and aCD47@PMSN for antibodies is essential in this work, and we found that the LC of aCD47@PMSN (14.2 ± 2.1%) increased significantly compared with aCD47@MSN (10.2 ± 1.9%) (Figure 3(e)). In addition, the encapsulation efficiency also increased from 51 ± 9.5% to 71 ± 10.5% (Figure 3(f)). The difference in the encapsulation efficiency (EE) and loading capacity (LC) is due to the secondary loading of antibodies during ultrasound and the loss of antibodies during centrifugation. First of all, in the process of platelet membrane coating mesoporous silicon nanoparticles, ultrasonic effect is beneficial to the secondary loading of nanoparticles to antibodies [49, 50]. Ultrasound helps more antibodies enter the mesopore of the MSN. Thus, there are more antibodies loading into the final product-B aCD47@PMSN in the tube B. Furthermore, in the tube A, the antibodies in the final product-A aCD47@MSN are less than the antibodies in the final product-B aCD47@MSN as the antibodies are thrown out by centrifugal force during the centrifugal process. In the tube B, the platelet membrane encapsulation can reduce the loss of antibodies during centrifugation. In some previous related work, a similar trend can be seen. For instance, Ge et al. studied platelet membrane-coated PLGA nanoparticles loaded with rapamycin; there was also an ultrasound time of 2 minutes during the preparation of platelet membrane-coated PLGA nanoparticles; the LC and EE of rapamycin were increased by platelet membrane encapsulation [38]. Wang et al. wrapped RBC membranes onto the surface of PLGA nanoparticles loaded rapamycin, also involving an ultrasound time of 2 min during the preparation of RBC membrane-coated PLGA nanoparticles; the LC and EE of rapamycin were also increased by RBC membrane encapsulation [15]. Therefore, it is not unusual to find a lower residual antibody concentration in the supernatant (tube B), so it is not surprising that the encapsulation efficiency (EE) and loading capacity (LC) of aCD47@PMSN are higher.

Furthermore, anti-CD47 antibody release was tested in vitro to check whether coating platelet vesicles on MSN affects the drug release profile. The release curve has no burst phase, suggesting that MSN encapsulated in platelet vesicles effectively prevented the leakage and loss of antibodies.

The release rate of anti-CD47 in PMSN was slightly lower than that in MSN, indicating that the platelet membrane coating had only minor effect on the antibody release (Figure 3(g)).

### 3.2. aCD47@PMSN Nanoparticles Display Immune-Evasive Properties In Vitro and In Vivo

The key proteins on the surface of platelet membrane play a decisive role in its function of immune escape and targeting atherosclerosis. Thus, protein integrity after PMSN fabrication was demonstrated. SDS-PAGE revealed that almost all membrane proteins were well retained throughout the PMSN fabrication process (Figure 3(h)). The ability of the platelet membrane to escape macrophage recognition is attributed to the synergistic effect of various functional membrane proteins on their surface. CD47, widely expressed on the surface of the platelet membranes, plays a key role in regulating phagocytosis by macrophages by bonding with the SIRP-*α* receptor. Therefore, CD47 expression on platelet vesicles and PMSN was tested using western blot analysis. The results clearly showed that the presence of CD47 on PMSN was almost the same as it on platelets (Figure 3(i)).

Thereafter, we evaluated the immune escape ability of PMSN by studying the endocytosis kinetics of macrophages, via incubating PMSN-RITC and MSN-RITC with RAW 264.7 cells. Image observation by CLSM verified that with the increasing coculture time, the red fluorescence in macrophages became stronger and stronger, indicating that the phagocytosis of MSN by macrophages increased with time. In contrast, there was still no red signal in the macrophages in the PMSN group after 12 h of coculture, indicating that PMSN could effectively evade phagocytosis by macrophages (Figure 3(j)).

Compared with unmodified nanoparticles, cell membrane-coated nanoparticles have been reported to show longer blood circulation time. To verify this conclusion, we studied the pharmacokinetics of MSN and PMSN in adult male balb/c mice. After tail vein injection of MSN-RITC or PMSN-RITC, blood was drawn at a predetermined time interval from the tail vein to measure the fluorescence intensity. Compared with the rapid clearance of MSN after injecting for 4 h, the PMSN-RITC nanoparticles exhibited about 31.8% and 18.3% overall retention in blood after 24 and 48 h (Figure 3(k)). These results showed that the platelet membrane coating indeed prolonged the circulation time of the nanoparticles.

### 3.3. In Vitro and In Vivo Target Atherosclerotic Plaque

In order to test the NPs' ability of targeting plaque, we checked the binding affinity and binding kinetics of PMSN and MSN in vitro under different shear stress. The result indicated that PMSN can effectively accumulate subcutaneously in the injured vessel under the 4 to 8 dyne/cm^2^ shear stress. As shown in Fig S6 and Fig S7, under the same shear stress, the PMSN can effectively accumulate subcutaneously more than MSN. Furthermore, as the shear stress increases, so does the amount of accumulation. The strong signal of PMSN observed by Ex Vivo FL Imaging and tissue sections demonstrates that PMSN can effectively attach and enter the plaque under shear stress range from 4 to 8 dyne/cm^2^.

**Figure 4:**
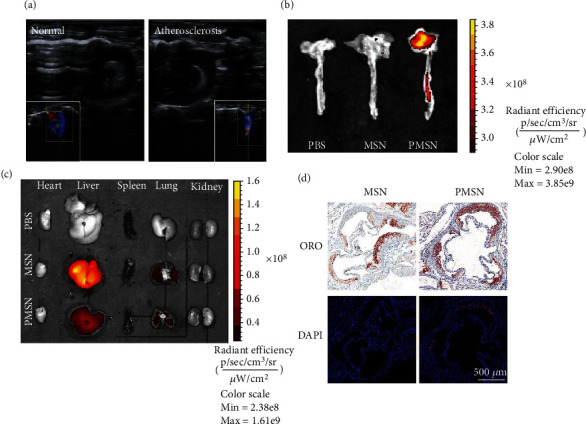
(a) The ultrasound image of aortic arch in normal mice and ApoE^−/−^ mice with atherosclerosis. (b) Accumulation of MSN and PMSN in atherosclerotic plaques of ApoE^−/−^ mice observed by Ex Vivo FL Imaging. (c) Accumulation of MSN and PMSN in the heart, liver, spleen, lung, and kidney of ApoE^−/−^ mice observed by Ex Vivo FL Imaging. (d) Tissue sections of atherosclerotic plaques in ApoE^−/−^mice injected with MSN and PMSN (blue: DAPI; red: MSN-RITC and PMSN-RITC).

After 8 weeks of western-type diets, atherosclerosis induced in the ApoE^−/−^ mice was confirmed by sonography. Ultrasound images of normal mice (Figure 4(a)) show smooth walls of the aortic arch. However, in the ultrasound images of ApoE^−/−^ model mice, there was an uneven bulge in the vascular wall of the aortic arch and stenosis in the lumen, suggesting that atherosclerotic plaques were formed in the blood vessels of ApoE^−/−^ model mice. H&E and Oil Red O stained sections of the abdominal aorta showed obvious pathological lipid plaques in the blood vessel wall, which corroborated the sonography results, indicating that we successful formation of the atherosclerosis model. Immunostaining also demonstrated high expression of CD47 in the plaques, confirming the presence of the target proteins as reported in previous studies (Fig S8). [4]

In order to check the ability of PMSN in targeting atherosclerotic plaques, MSN-RITC and PMSN-RITC were intravenously injected into the atherosclerotic model mice. After 4 hours, the aorta was removed, and the concentration of nanoparticles in the aorta was observed by fluorescence imaging. As shown in Figure 4(b), almost no fluorescence signals were observed at the aortic arch in the MSN group, whereas significant fluorescence was determined in the PMSN group, demonstrating that PMSN did target the atherosclerotic plaque at the aortic arch.

In addition, we also measured the fluorescence signals in the heart, liver, spleen, lung, and kidney of the model mice. As shown in Figure 4(c), no obvious fluorescence was observed in the heart, spleen, and kidney in both PMSN group and MSN group. However, in the MSN group, obvious fluorescence signals were observed in the liver, stronger than that in the PMSN group. These results indicated that most MSN were cleared by the reticuloendothelial system (RES) of the liver [25].

In order to further observe the enrichment of PMSN targeted to plaques at the microscopic level, the tissue sections of atherosclerotic vessels were stained with Oil Red and DAPI, respectively. The images of atherosclerotic vessel sections were shown in Figure 4(d). In the PMSN group, a large amount of red fluorescence signals of PMSN-RITC were accumulated in the position corresponding to the atherosclerotic plaques in the Oil Red O tissue sections, while almost no red fluorescence signal was observed in the atherosclerotic plaques in the MSN group.

In conclusion, these results strongly demonstrated that PMSN could effectively and specially targeted atherosclerotic plaques and accumulated there, indicating that nanoparticles wrapped with platelet membranes retained the ability of immune escape and targeting atherosclerotic plaques as platelets.

### 3.4. In Vitro Efferocytosis Assay

**Figure 5:**
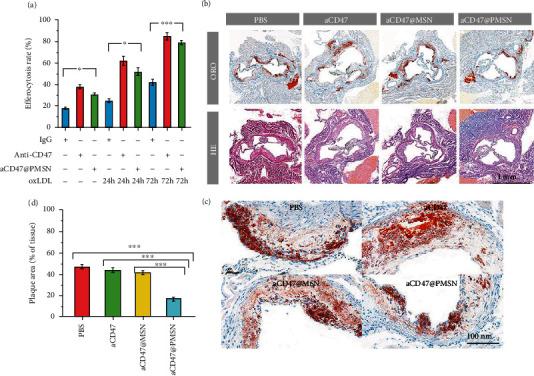
(a) Anti-CD47 antibody and aCD47@PMSN promote efferocytosis of vascular cells after exposure to a proatherosclerotic environment. (b) Pathological detections on the sections of aortic roots from ApoE^−/−^ mice after various treatments (Oil Red O and H&E) (all tissues: 200x). (c) A local enlarged view corresponding to (b) (Oil Red O) (all tissues: 1600x). (d) Quantitative data of the atherosclerotic plaque area in the aortic root sections (PBS: 46.7 ± 2%, aCD47: 43.5 ± 2.1%, aCD47@MSN: 41.6 ± 1.6%, aCD47@PMSN: 16.6 ± 1.7%). *n* = 5, mean ± SD, ^∗^*p* < 0.05, ^∗∗^*p* < 0.01, and ^∗∗∗^*p* < 0.001. ns: no significance.

We used an established in vitro phagocytosis assay to study whether anti-CD47 antibody could promote efferocytosis of smooth muscle cells after exposure to a proatherosclerotic environment. In this model, smooth muscle cells highly expressed CD47 after activation with TNF-*α*. We compared the effect of anti-CD47 antibody and aCD47@PMSN on promoting phagocytosis. The results of the flow cytometry analysis showed (Figure 5(a)) that the phagocytosis of smooth muscle cells was significantly increased to 63% and 85% by anti-CD47 in the groups exposed to the proatherogenic inflammatory environment for 24 h and 72 h, respectively. In addition, aCD47@PMSN also showed a certain role in promoting phagocytosis. It could also be found that the phagocytosis increased to 43% and 79%, respectively, in the 24 h and 72 h groups, both of which were significantly improved compared with the control group. Basically, aCD47@PMSN had slightly less effect on promoting phagocytosis than the pure anti-scheCD47 antibody, which might be ascribed to the retarded release of anti-CD47 antibody from the membrane-wrapped particles. In summary, in vitro model test demonstrated that aCD47@PMSN strongly promoted the phagocytosis of necrotic smooth muscle cells, implying that the granules also had the potential to promote the phagocytosis of necrotic smooth muscle cells in plaques in vivo.

### 3.5. Plaque Clearance In Vivo

The atherosclerotic ApoE^−/−^ mice were adopted to evaluate the antiatherosclerotic effect of the aCD47@PMSN in vivo. After 7 weeks of therapy (Figure S9), we acquired the aortas from the aortic arch to the iliac bifurcate to compare the therapeutic effect of atherosclerosis in different groups (PBS, free CD47 antibody, aCD47@MSN, aCD47@PMSN).

**Figure 6:**
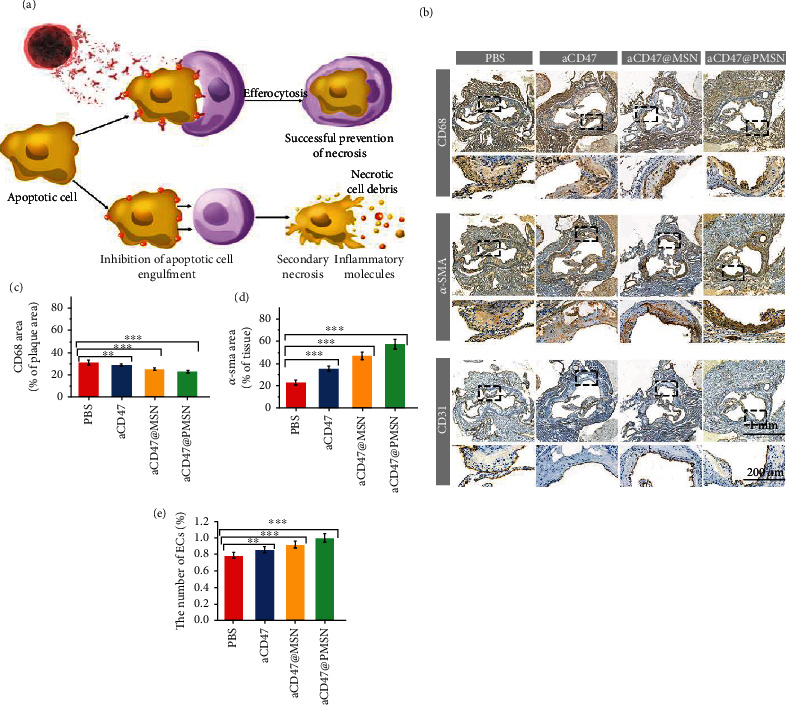
(a) Illustration of aCD47@PMSN promoted phagocytosis of necrotic smooth muscle cells. (b) IHC analysis of aortic roots from ApoE^−/−^ mice after various treatments (CD68, *α*-SMA, and CD31). (c) Quantitative data of the macrophages in the aortic root sections (PBS: 31.5 ± 1.8%, aCD47: 29.4 ± 1.3%, aCD47@MSN: 25.2 ± 0.8%, aCD47@PMSN: 23.3 ± 0.7%). (d) Quantitative data of the smooth muscle cells in the aortic root sections (PBS: 22.1 ± 2.3%, aCD47: 34.2 ± 2.1%, aCD47@MSN: 45.1 ± 2.9%, aCD47@PMSN: 55.2 ± 4.1%). (e) Quantitative data of the endothelial cells in the aortic root sections (PBS: 0.79 ± 0.03%, aCD47: 0.86 ± 0.04%, aCD47@MSN: 0.92 ± 0.04%, aCD47@PMSN: 1.01 ± 0.05%). *n* = 5, mean ± SD, ^∗^*p* < 0.05, ^∗∗^*p* < 0.01, and ^∗∗∗^*p* < 0.001. ns: no significance.

Through the Oil Red O staining of the cross-sections (Figures 5(b) and 5(c)), it could be intuitively found that, compared with the control group, the plaque area of the aCD47 and aCD47@MSN groups decreased (from 46.7 ± 2% to 43.5 ± 2.1% and 41.6 ± 1.6%, respectively), and the average plaque area of the aCD47@PMSN group decreased even more (16.6 ± 1.7%) (Figure 5(d)). The H&E stained images showed that the control group had more acellular lipid cores and cholesterol crystals, while the aCD47@PMSN group had the least lipid cores and almost no cholesterol crystals. All the above results directly indicated that aCD47@PMSN effectively reduced the area of the atherosclerotic plaque and cleared the necrotic lipid core of the plaque (Figure 6(a)). Moreover, the therapeutic effect of the aCD47@PMSN group was better than that of the aCD47@MSN group and the aCD47 group. The main reason was that the PMSN nanoparticles were coated with platelet membrane, allowing the nanoparticles to evade recognition by the immune system and phagocytosis by macrophages, giving it a longer circulatory half-life. At the same time, the coating of the platelet membrane gave the particle the ability to target atherosclerotic plaques, which enabled more nanoparticles to accumulate in the plaques.

Next, we analyzed the composition of atherosclerotic plaque in the aortic root sections by immunohistochemistry. Macrophages play a key role in the initiation and progression of atherosclerotic inflammation. As shown in Figures 6(b) and 6(c), the CD68 protein staining results showed that the aCD47@PMSN group had the least macrophages in the atherosclerotic plaques, compared with the other three groups. The released anti-CD47 antibody from aCD47@PMSN blocked the highly expressed CD47 protein on the surface of the necrotic cells in the plaques so that macrophages could identify and engulf the necrotic cells in the plaque. The timely removal of the necrotic cells reduced the inflammation in the plaque.

As shown in the *α*-SMA stained sections (Figure 6(b)), the distribution of smooth muscle cells in atherosclerotic plaques was also different among each group. In the aCD47@PMSN group, the surface of the small plaques had the most smooth muscle cells, while that was the least in the control group (Figure 6(d)). More smooth muscle cells on the plaque surface lead to thicker fibrous caps and more stable plaques, while thinner fibrous caps indicate that the plaques are more likely to rupture and cause thrombosis [6, 51]. It indicated that after aCD47@PMSN treatment, the smooth muscle cells on the surface of the plaque were protected while the plaque area was reduced, and the inflammation decreased. This caused the formation of a thick fibrous cap, stabilizing the plaques and reducing the risk of thrombosis by plaque damage.

For atherosclerotic plaques, in addition to the growth of the core rich in necrotic cells and lipids, the continuous inflammatory reaction also damages the endothelial cell layer on the surface of plaques, which is also the direct cause of thrombosis in advanced stages of atherosclerotic plaques. Damage to the plaque endothelium exposes the structure inside the plaque, and the necrotic cells, lipid core, and type iv collagen activate blood platelets, increasing the risk of thrombus formation, finally occludes the blood vessel and causes an infarction. Therefore, immunohistochemical staining of CD31 protein was also used to detect the endothelial cells on atherosclerotic plaques after the different treatments. In the control PBS group, the CD31 signal was significantly less than in the aCD47@PMSN group, indicating that the overall inflammation of the plaques and other lesions was improved through our treatment regimen (Figures 6(b) and 6(e)). This could alleviate further deterioration and reduce the damage to the endothelial cells. These results demonstrated that while reducing the area of atherosclerotic plaque, aCD47@PMSN might also improve the function of the plaque endodermis, preventing plaque damage and reducing the risk of thrombosis.

### 3.6. Biocompatibility Test

The biocompatibility of aCD47@PMSN was investigated after four-week treatment. There was no significant difference in body weight of the mice between the various treated groups. Moreover, no obvious difference in the hematoxylin-eosin (H&E) section of the heart, liver, spleen, lung, and kidney was observed, suggesting no significant toxicity to the main organs. These results showed that PMSN had good biocompatibility and no obvious toxic and side effects (Fig S10).

## 4. Conclusion

In summary, we developed platelet membrane-coated MSN as a smart delivery system for targeted delivery of anti-CD47 antibody to atherosclerotic sites. The biomimetic nanoparticles (PMSN) could mimic the platelet to escape the immunoclearance in the reticuloendothelial system and specifically target the atherosclerotic plaques, accumulate in the necrotic cores, and release the antibody. Compared with the unmodified nanoparticles (MSN), PMSN showed the ability to escape the macrophage recognition in vitro and had longer circulating half-life in vivo. In ApoE^−/−^ mice with atherosclerosis, PMSN specifically accumulated in atherosclerotic plaques. Compared with free anti-CD47 antibody, aCD47@PMSN significantly prevented the necrotic decay of cells but significantly promoted the efferocytosis of necrotic cells in the plaques. Clearing the dead cells greatly reduced the atherosclerotic plaque area, stabilized plaques, and reduced the risk of plaque rupture and advanced thrombosis. No significant side effects were observed in mice, indicating the desirable safety of PMSN.

Overall, our work demonstrated that the platelet membrane-coated MSN loading anti-CD47 antibody was an effective treatment strategy for atherosclerosis. PMSN appear as the new class of safe and effective target drug delivery system for cardiovascular diseases, and even for other diseases like cancer, emerging a high potential for clinical translation.

## Data Availability

The data used to support the findings of this study have not been made available online because some of them are essential data for further study. The data are available from the corresponding authors upon reasonable request.

## Supplementary Materials

Supplementary Materials Figure S1: schematic diagram of preparation process of aCD47@MSN (tube A) and aCD47@PMSN (tube B). Figure S2: pore size distribution of MSN (provided by the supplier Xi'an ruixi Biological Technology Co., Ltd.). Figure S3: morphology of platelet membrane vesicle observed by TEM (scale bar left: 50 nm; right: 500 nm). Figure S4: morphology of nanoparticles observed by TEM. Top: MSN; bottom: PMSN (scale bar = 50 nm, 100 nm). Figure S5: the TEM image of nanoparticles stained by a negative staining technique. Top: MSN; bottom: PMSN (scale bar = 50 nm, 100 nm). Figure S6: accumulation of MSN and PMSN in the injured femoral artery of New Zealand Rabbit under different shear stress in vitro observed by Ex Vivo FL Imaging. Figure S7: tissue sections of injured femoral artery of New Zealand Rabbit (blue: DAPI; red: MSN-RITC and PMSN-RITC; green: CD31). Figure S8: IHC analysis of atherosclerosis in the ApoE^−/−^ mice after 8 weeks of western-type diets (HE, ORO, CD68, and CD47) (all tissue: 200x). Figure S9: scheme of treating plan for in vivo therapeutic study. Figure S10: H&E images of tumors and major organs after different treatments for 1 month (all tissue: 200x).
