# Supplementary material for: Platelet Membrane-Coated Nanocarriers Targeting Plaques to Deliver Anti-CD47 Antibody for Atherosclerotic Therapy
Source: Research (Wash D C). 2022 Jan 17;2022:9845459. doi: 10.34133/2022/9845459 (PMC8791388; doi:10.34133/2022/9845459)
Supplement: Supplementary Materials — Figure S1: schematic diagram of preparation process of aCD47@MSN (tube A) and aCD47@PMSN (tube B). Figure S2: pore size distribution of MSN (provided by the supplier Xi'an ruixi Biological Technology Co., Ltd.). Figure S3: morphology of platelet membrane vesicle observed by TEM (scale bar left: 50 nm; right: 500 nm). Figure S4: morphology of nanoparticles observed by TEM. Top: MSN; bottom: PMSN (scale bar = 50 nm, 100 nm). Figure S5: the TEM image of nanoparticles stained by a negative staining technique. Top: MSN; bottom: PMSN (scale bar = 50 nm, 100 nm). Figure S6: accumulation of MSN and PMSN in the injured femoral artery of New Zealand Rabbit under different shear stress in vitro observed by Ex Vivo FL Imaging. Figure S7: tissue sections of injured femoral artery of New Zealand Rabbit (blue: DAPI; red: MSN-RITC and PMSN-RITC; green: CD31). Figure S8: IHC analysis of atherosclerosis in the ApoE−/− mice after 8 weeks of western-type diets (HE, ORO, CD68, and CD47) (all tissue: 200x). Figure S9: scheme of treating plan for in vivo therapeutic study. Figure S10: H&E images of tumors and major organs after different treatments for 1 month (all tissue: 200x). [file 9845459.f1.docx]

Supporting Information

**Platelet Membrane-Coated Nanocarriers Targeting Plaques to Deliver anti-CD47 Antibody for Atherosclerotic Therapy**

Liang Chen^a, 1^, Zhongyi Zhou^a, 1^, Cheng Hu^a^, Manfred F. Maitz^b, c^, Li Yang ^a^, Rifang Luo*^, a^, Yunbing Wang*^, a^

^a^ National Engineering Research Center for Biomaterials, Sichuan University, Chengdu 610065, China

^b^ Max Bergmann Center of Biomaterials, Leibniz Institute of Polymer Research Dresden, Dresden, 01069, Germany

^c^ Key Lab. for Advanced Technologies of Materials, Ministry of Education, School of Material Science and Engineering, Southwest Jiaotong University, Chengdu, 610031, China

^1^ These authors contributed equally.

* Corresponding author

E-mail address: lrifang@scu.edu.cn (R. Luo), [yunbing.wang@scu.edu.cn](mailto:yunbing.wang@scu.edu.cn) (Y. Wang).


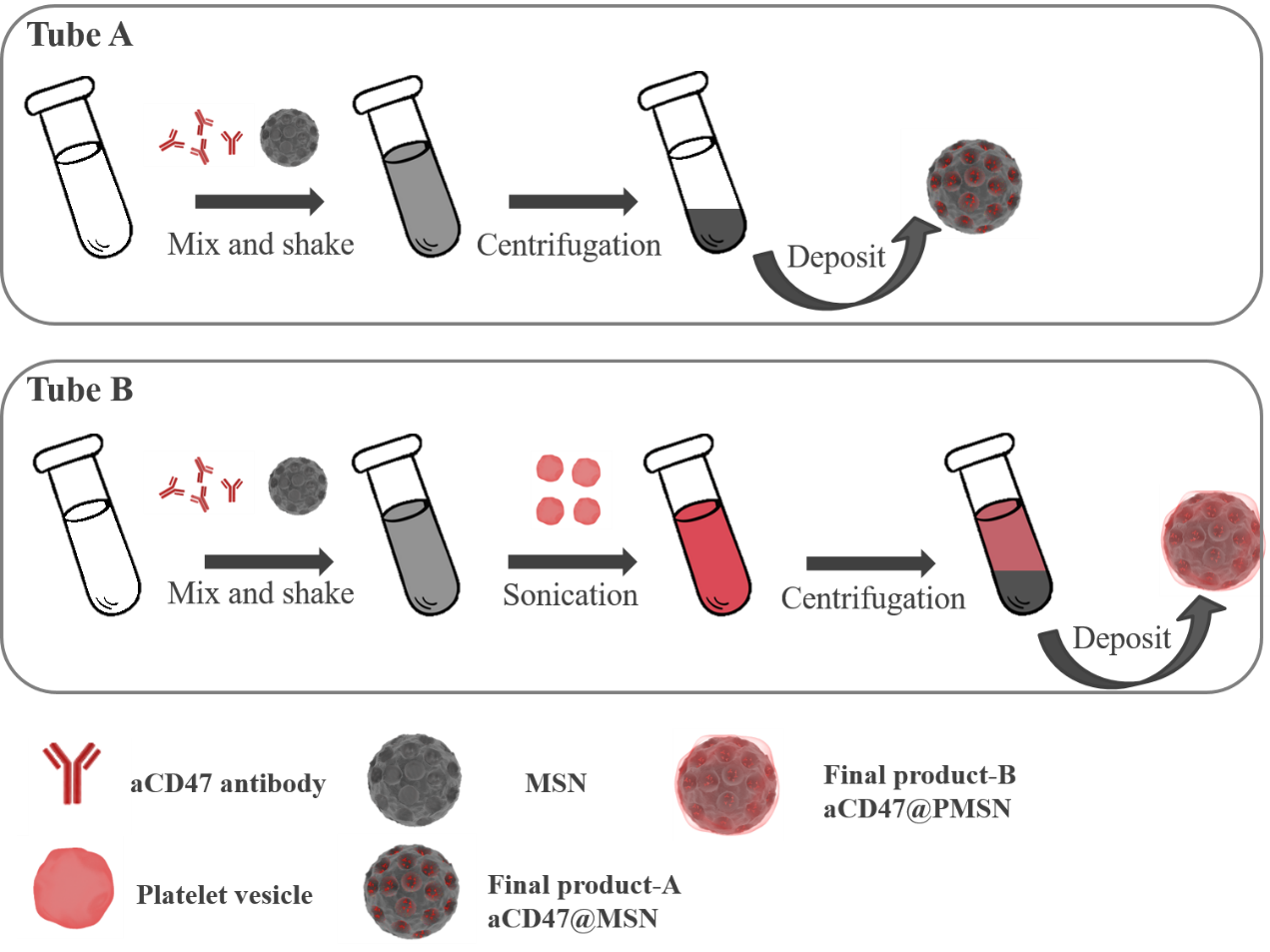


**Figure S1.** Schematic diagram of preparation process of aCD47@MSN (Tube A) and aCD47@PMSN (Tube B).


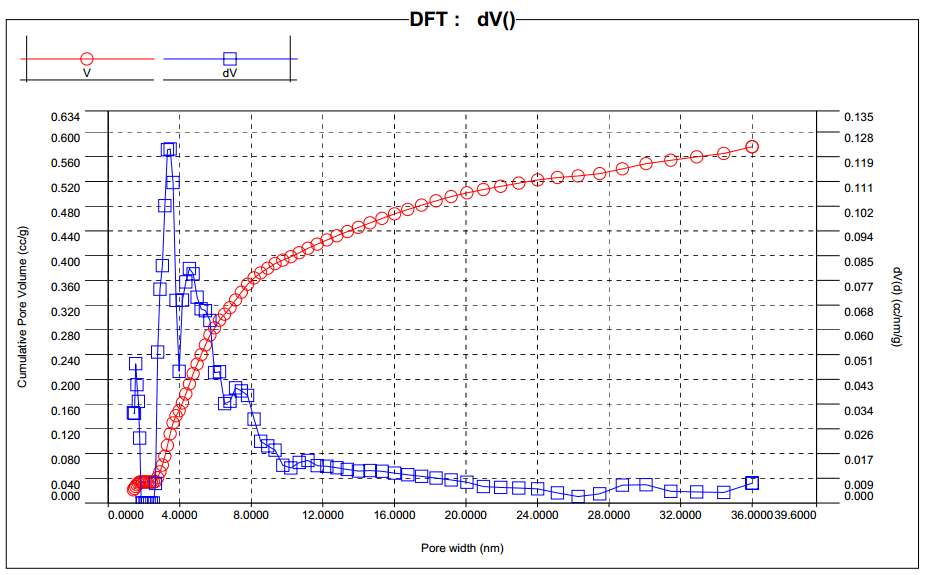


**Figure S2.** Pore size distribution of MSN (provided by the supplier Xi'an ruixi Biological Technology Co.,Ltd)


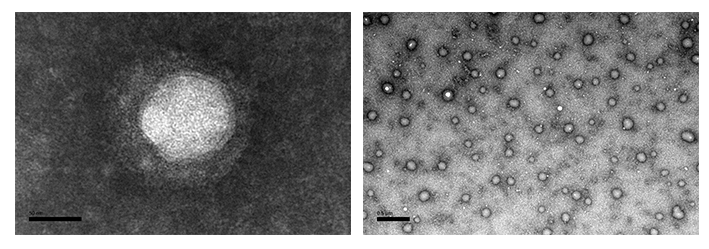


**Fig S3.** Morphology of Platelet membrane vesicle observed by TEM. (Scale bar Left: 50 nm; Right: 500 nm).


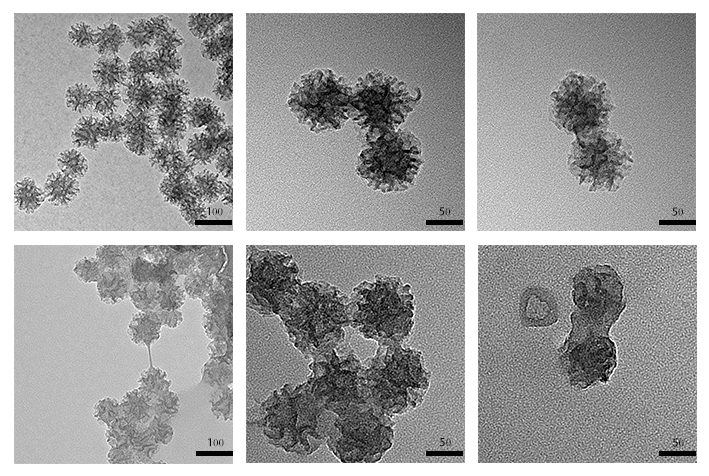


**Fig S4.** Morphology of nanoparticles observed by TEM. Top: MSN; bottom: PMSN (scale bar = 50 nm, 100 nm).


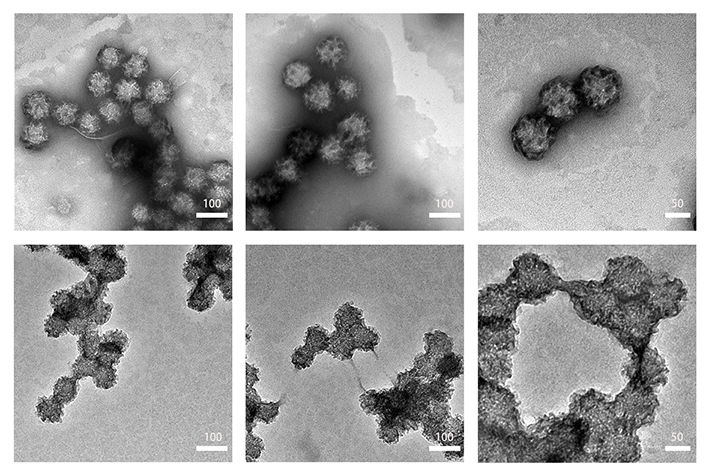


**Fig S5.** The TEM image of nanoparticles stained by a negative staining technique. Top: MSN; bottom: PMSN (scale bar = 50 nm, 100 nm).


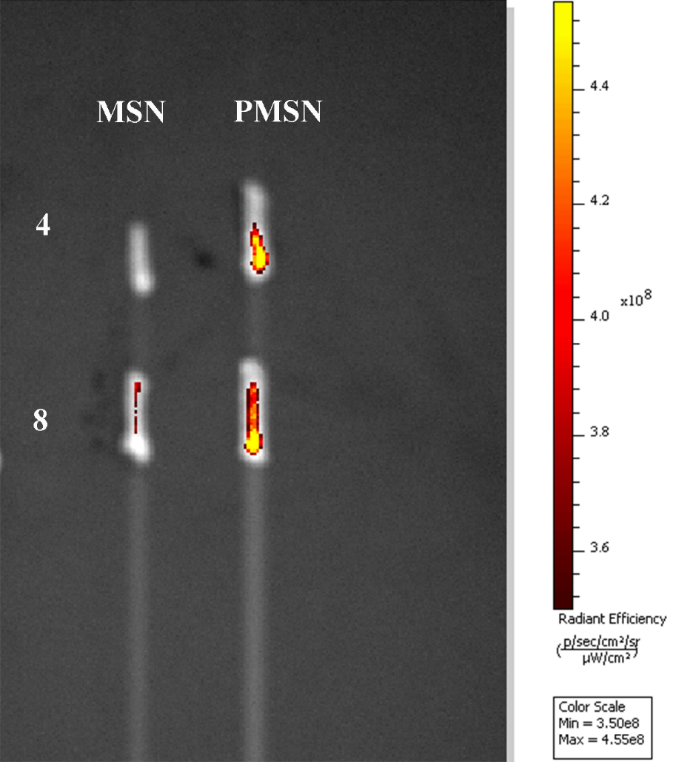


Figure S6. Accumulation of MSN and PMSN in the injured femoral artery of New Zealand Rabbit under different shear stress in vitro observed by Ex Vivo FL Imaging.


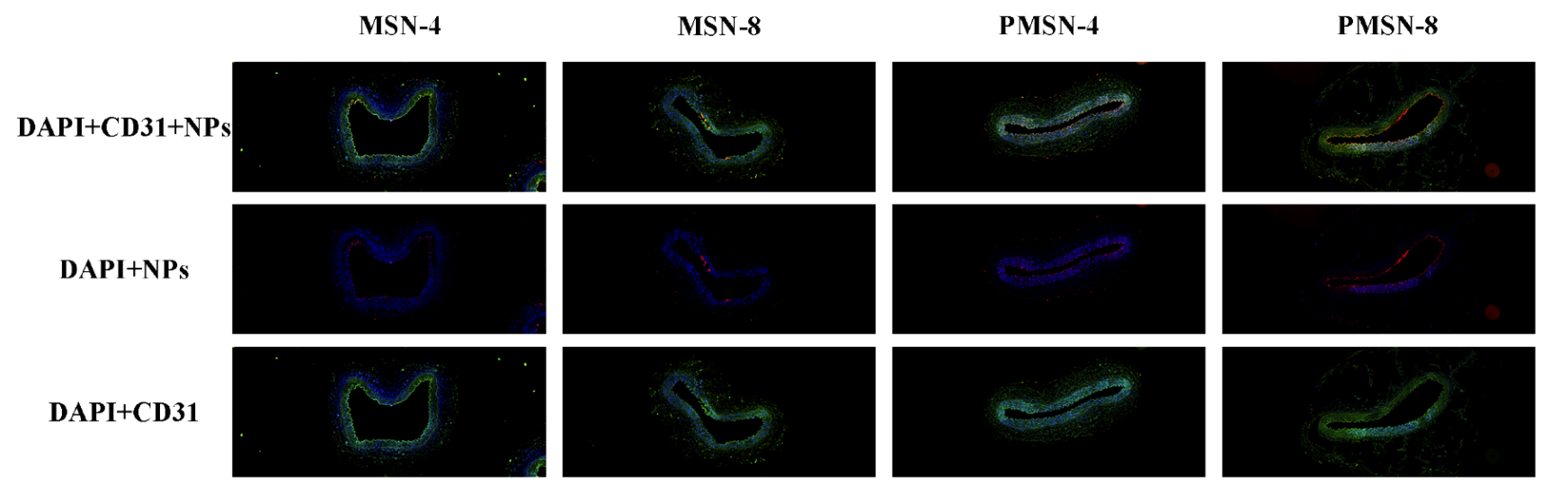


Figure S7. Tissue sections of injured femoral artery of New Zealand Rabbit. (Blue: DAPI; Red: MSN-RITC and PMSN-RITC; Green: CD31).


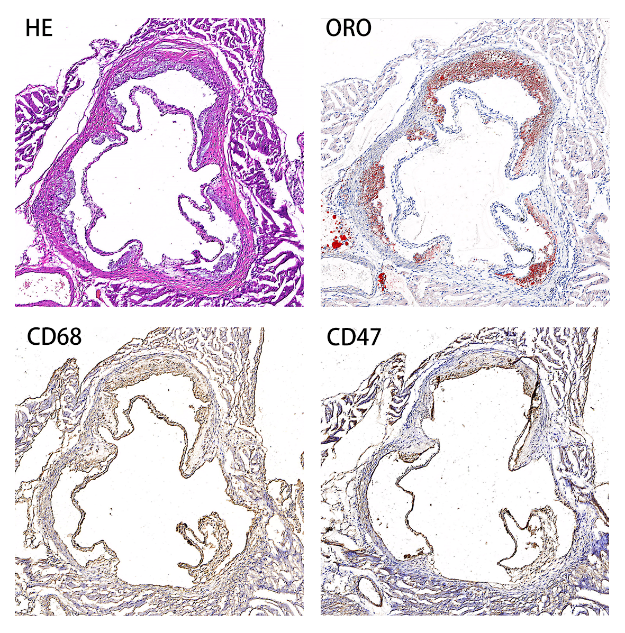


**Fig S8.** IHC analysis of atherosclerosis in the ApoE^−/−^ mice after 8 weeks of western-type diets. (HE, ORO, CD68, and CD47) (all tissue: 200×).


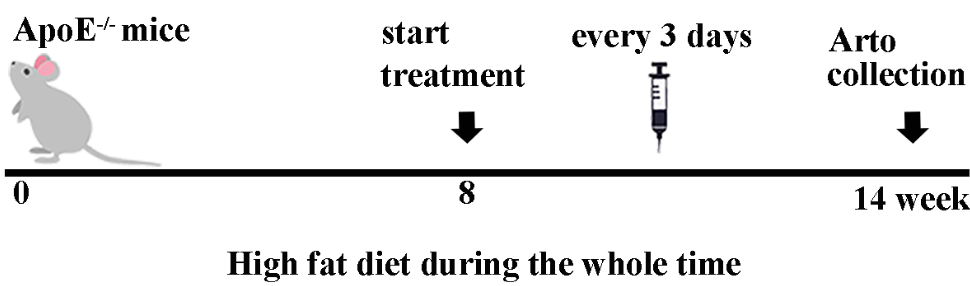


**Fig S9.** Scheme of treating plan for *in vivo* therapeutic study.


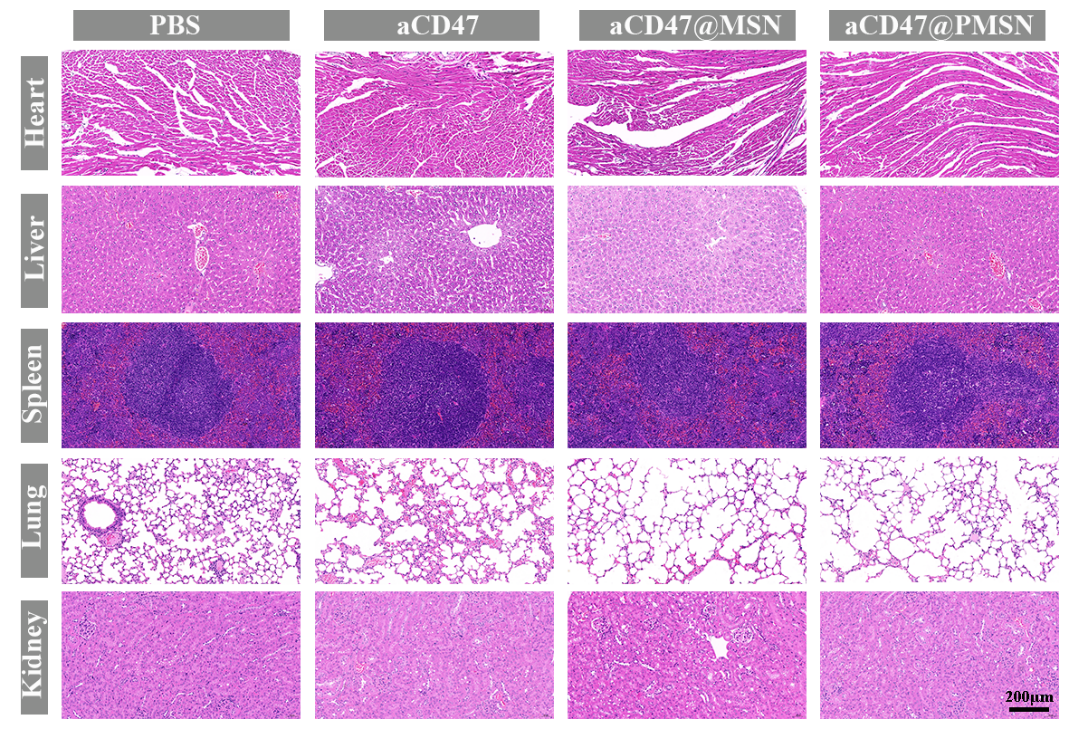


**Fig S10.** H&E images of tumors and major organs after different treatments for 1 month (all tissue: 200×).
